# Webcam technology on neonatal wards—examining the objective and subjective workload of nurses: a combined observational and survey study

**DOI:** 10.1186/s12912-024-02107-4

**Published:** 2024-07-02

**Authors:** Helena Sophie Müller, Michael Becker-Peth, Ludwig Kuntz, Neo-CamCare, Nadine Scholten, Andreas Müller, Till Dresbach, Martin Hellmich, Christina Samel, Christiane Woopen, Christiane Jannes, Ludwig Kuntz, Indra Spiecker gen Döhmann, Sebastian Bretthauer, Dirk Horenkamp-Sonntag, Stefanie Wobbe-Ribinski

**Affiliations:** 1 Department of Business Administration and Health Care Management, Faculty of Management, Economics and Social Sciences, University of Cologne, Cologne, Germany; 2 Department of Technology and Operations Management, Rotterdam School of Management, Erasmus University, Rotterdam, The Netherlands

**Keywords:** Workload, Webcam technology, Nurse, Neonatology

## Abstract

**Background:**

This study was conducted to estimate the additional objective and perceived workload of nurses resulting from the use of webcams. The successful implementation of webcam technology into routine care requires an analysis to prevent adverse events of increased nursing workload.

**Methods:**

The study took place on three neonatal wards in two University Hospitals in Germany. In the first Hospital, the study was conducted from February to July 2021; in the second one it was conducted between June and November 2021. Data were collected using a combined approach of a standardised diary questionnaire study and passive observations. The participants were accompanied in their daily work and their activities were recorded 65 nurses participated.

**Results:**

2,031 h were observed in 1,630 observation blocks. In 14.74% of the observation blocks webcam activities were detected. The extent to which the nurses had webcam-related additional workloads was rated as no additional workload in 82.16% of the daily questionnaires (*n* = 1,026).

**Conclusion:**

The observed low workload due to the webcams is in line with the nurses’ perception. The observational data revealed, on a number of different analysis levels, that a limited additional workload was generated. There was no decrease in activity performance observed and no clear indication for interruptions due to the webcam-related activities for the nurses. However, it is important to raise awareness about the individual workload levels for the successful implementation. Additional education programs can be provided for nurses.

**Trial registration:**

The Neo-CamCare study is registered at the German Clinical Trials Register. DRKS-ID: DRKS00017755.

**Supplementary Information:**

The online version contains supplementary material available at 10.1186/s12912-024-02107-4.

## Background

The implementation of new technologies in hospitals can have a variety of effects on the work process for nurses. Telemedical innovations could offer new approaches to optimise or simplify care [1], but the technical infrastructure and the willingness of the staff must also be given for successful technology implementation.

One possibility for the implementation of telemedicine in the hospital is webcam technology on neonatal wards. Preterm infants with a low birth weight often have to be cared for in hospital for a long period of time and lie in incubators. This separation of premature infants and parents and the long hospital stay of the child can have a wide variety of negative consequences. Parents are confronted with various stress factors due to the setting, which increases their stress level and can trigger anxiety [2, 3].

The use of webcam technology allows parents to observe their child in the incubator via live stream from home. Particularly during the Covid-19 pandemic, when visiting hours in hospitals often had to be restricted, the technology became even more important [4].

In previous studies, various positive effects of the webcam technology from the parental perspective could be observed. The use of webcams reduced, for example, the parents’ feelings of stress and anxiety and improved the parental feeling of reassurance [1, 5–7] Weber et al. [8] observed a positive influence of the webcam with regard to breastmilk-feeding. In a recent study conducted by Chant et al. [9] no change or a decrease in anxiety levels for the majority of interviewed parents were identified. However there might be also fears and concerns of parents regarding the webcam use, which need to be addressed in the care process [10]. Overall, the use of webcam technology was associated in multiple studies with different positive benefits from the parental perspective [11].

For a widespread and long-term implementation of the technology, the evaluation of the extent to which the webcams lead to an additional workload for the nursing staff is essential, since the nurses on the ward are mainly responsible for the implementation of the technology. They turn the webcams on and off and often take care of the communication with the relatives [7, 12]. The setting of the neonatal intensive care unit provides care for pre-term and new-born infants and is associated with demanding work situations for nurses like life-saving actions or the care of ventilator-dependent child [13]. Therefore, it is important to analyse how much time nurses spend on the webcams during their shifts and whether the technology can be integrated into the workflow to prevent possible adverse events of increased workload [14–17]. This article contributes by measuring the objective and subjective workload of the nurses through the implementation of webcam technology.

In preceding studies, there was conflicting evidence regarding the impact of webcams on nurses’ work. Joshi et al. [7] ascertained in their earlier study that nurses spent a significant amount of time implementing the webcam, as well as communicating with parents about the technology. In their questionnaire-based study, Joshi et al. [7] found that these webcam-related actions caused disruption in the nursing workflow.

More recent evidence from Chant et al. [18] identified no negative impact on nursing workload. Kilcullen et al. [19] analysed on a single neonatal ward the staff perception of webcam use and identified concerns regarding the workload and technical problems. Another study did not find an association that the technology caused additional stress for nurses [6]. Furthermore, a rather critical attitude of the nurses towards the webcam technology was evaluated. The interviewed nurses feared that webcams could increase the stress level for nurses and parents [5, 6].

These conducted studies assessed the nurses’ perspective and webcam-related workload through qualitative and quantitative questionnaires. In order to evaluate the extent to which the nurses’ perceptions correspond to the actual workload caused by the webcams, further analyses are necessary. The aim of this study was to record the actual, objective workload caused by the implementation of the technology and the subjective perception of the nurses’ workload. We analysed potential interruptions, observed activities and the distribution of webcam activities across different shifts to investigate whether the technology was contributing to an increased workload for nurses. Given the results from the observation data, we considered the nurses' subjective assessment of workload, in order to address the research question of how the integration of webcams within neonatal wards impacted nursing workload.

## Methods

The data were collected as part of the Neo-CamCare study. The study group of Neo-CamCare was divided into various work packages and followed a mixed-methods-approach containing different forms of data collection and focusing on different outcomes [20]. Neo-CamCare has been registered in the German Register of Clinical Studies (ID: DRKS00017755). Our work package used a study design that combines an observational study with a standardised diary questionnaire study during the same time period. The outcomes were independently assessed from other work packages of the Neo-CamCare study. The study took place on three neonatal intensive care units in two University Hospitals in Germany.

In the first Hospital, the study was conducted on two wards from 1 February 2021 to 31 July 2021; and in the second one it was conducted between 1 June 2021 and 30 November 2021. All nurses working on the wards (*n* = 171) were invited to participate. 65 nurses participated in the study. Informed consent was obtained from each participant.

### Observation

The observation of the additional webcam-related workload for the nurses was collected in a passive observational study using a methodology based on those of Langhammer et al. and Sülz et al. [21, 22]. They assessed the workload of nurses through passive observation. For this purpose, observation intervals of a total of 120 min per observation day were utilized [21]. In order to ensure sufficiently time intervals during which webcam activities can take place, the observation block time in our study was set from 1h15 min to 1h30 min per nurse. During the study period, the participating nurses on the wards were accompanied in their daily work, and their activities were recorded in observation blocks. The nurses participating in the study were observed by students, who studied medicine, health economics or nursing sciences. Thus, a basic understanding of medical processes and nursing activities can be assumed. At the beginning of the observation period, a digital workshop informed them about the study-related activities and content. In addition to that, they received the same manual providing guidance on the study and recording of the activities. The observers used tablet PCs and a self-modified software application to record the nursing activities passively. The observers accompanied the nurses during their work and observed all tasks passively. Through this methodology, several different nurses could be observed during their shifts on one day on the ward. At the beginning of each shift, the observers recorded the pseudonyms of the nurses who had given their consent to participate. Subsequently, a random observation sequence was generated by the software application. The observers recorded, for each activity, the exact starting time and end of the activities. The activities were classified into categories with a code assigned to each category (direct care, indirect care, administration, webcam-related activities, care of another child (each nurse gets patients assigned at the beginning of the shift, if they perform activities on other patients, this code was selected) and other). The taxonomy of nursing tasks was based on Milligan et al. [23] and Pillay et al. [24]. It was modified for the German context and the webcam related activities were added for the study purpose. The task taxonomy was implemented in the software application to simplify the selection of the correct task category for the observers. If the observers were not confident with a selected coding, they could indicate this by adding a comment. The code for a webcam-related activity was always selected by the observers when the nurse was directly involved with the webcams. This includes switching the webcams on and off, as well as adjusting them or solving technical problems. Communication with relatives of the patients regarding the webcams was also recorded as a webcam-related activity.

### Questionnaires

At the beginning of the study, the participating nurses filled out a general questionnaire about demographic characteristics. If the participants were accompanied by an observer during data collection, they were asked every time to complete a questionnaire on the same observation day about the webcam-related additional workload. We developed the webcam related questions for the study (see Supplementary file 1) and pretested the questionnaire with nurses (*n* = *5*). The nurses completed the questionnaire and provided feedback regarding the comprehension of the items and the overall questionnaire structure. 4-point item scales were used to assess the perceived additional workload (0 [no additional workload]; 1 [rather low]; 2 [rather high]; 3 [very high]).The internal reliability was assessed using Cronbach’s alpha (α = 0.81). For participating in the study, the nurses received 30€. As an incentive to complete the daily questionnaires over the study period, they received another payment of 10€ per 10 completed questionnaires.

### Statistical analysis

StataMP 17 (StataCorp LLC.) was used to analyse the observation and questionnaire data. The observers had the opportunity to add comments if they were not confident with the selected coding. Accordingly, all comments were checked and the data were cleaned before the analysis. Descriptive statistics were used to describe the data and answer the research question. In order to examine whether the questionnaires could be analysed on aggregate, an ANOVA was conducted.

At one University Hospital, the observation started on 1 February 2021, but in the first two weeks, there were technical problems with the tool. Therefore, only data from 15 February to 31 July were included in the analysis.

## Results

### Observation data

A total of 65 nurses participated in the study. The participation rate in the study was 38.01%. Table 1 presents the characteristics of the study sample. The average age of all participating nurses was 32.41 years, with the youngest nurse being 21 years old and the oldest nurse being 61 years old. The majority of the nurses was female (96.92%), 26 to 35 years old (50.77%) and worked between one and five years in pediatric nursing (41.54%). For the age structure, a comparison of all nurses on the wards was possible; the age group of 18 to 25 years was proportionately more represented in the study sample.

**Table 1:** Sample characteristics (*n* = 65)

	Study sample (*n* = 65)	%	All nurses (*n* = 171)	%	Absolute difference (in %)
**Age**					
18 to 25 years	14	21.54	18	10.53	+ 11.01
26 to 35 years	33	50.77	92	53.80	-3.03
36 to 45 years	11	16.92	32	18.71	-1.79
46 to 55 years	5	7.69	19	11.11	-3.42
56 to 65 years	2	3.08	10	5.85	-2.77
**Gender**					
Female	63	96.92			
Male	2	3.08			
**Years working in pediatric nursing**					
Less than 1 year	6	9.23			
1 to 5 years	27	41.54			
6 to 10 years	11	16.92			
More than 10 years	21	32.31			
**Years working on the ward**					
Less than 1 year	14	21.54			
1 to 5 years	30	46.15			
6 to 10 years	9	13.85			
More than 10 years	12	18.46			

During the study period, the observers recorded 2,031 h, distributed across 1,630 observation blocks. One block lasted on average 74.77 min. (median = 75.82 min.; modus = 75.13 min.; range = 0.2—139.52 min.). The block length varied because the observers adapted the passive observation to the nurses’ workflow in order to avoid abrupt termination of the block. 41.84% of the blocks took place in the early shift, 41.72% in the late shift and 16.44% in the night shift (the early shift lasted from 6 a.m. to 3 p.m., the late shift lasted from 1 p.m. to 8 p.m. and the night shift lasted from 8 p.m. to 7 a.m.).

Over the study period, a total of 333 webcam-activity codes were identified. 0.91% of all observed activities were webcam activities. The webcam related activities were distributed over 166 observation blocks. Out of the 1,630 blocks, 504 blocks did not include children with a webcam attached, because only a subset of bed had a webcam attached, and a random sample of nurses were observed. This results in 1,126 webcam observation blocks and 504 non-webcam observation blocks. The share of blocks with webcam activities was 14.74%, excluding these 504 blocks where no webcam was attached to the incubator and therefore no webcam activity was possible.

In 78.92% of the webcam observation blocks, a maximum of two webcam activities were observed. In order to be able to evaluate the amount of work caused by the webcam system, the duration of the webcam activities is relevant. 3.93 h of webcam activities were identified in total during the study period. The average duration of one webcam activity is 42.51 s. 49.85% of all observed webcam activities lasted a maximum of 20 s. The maximal duration was 12 min. The number of webcams per observation block resulted from the availability of webcams on the wards and the number of patients with a webcam (e.g. depending on how many parents agreed to use the webcam).

**Fig. 1:**
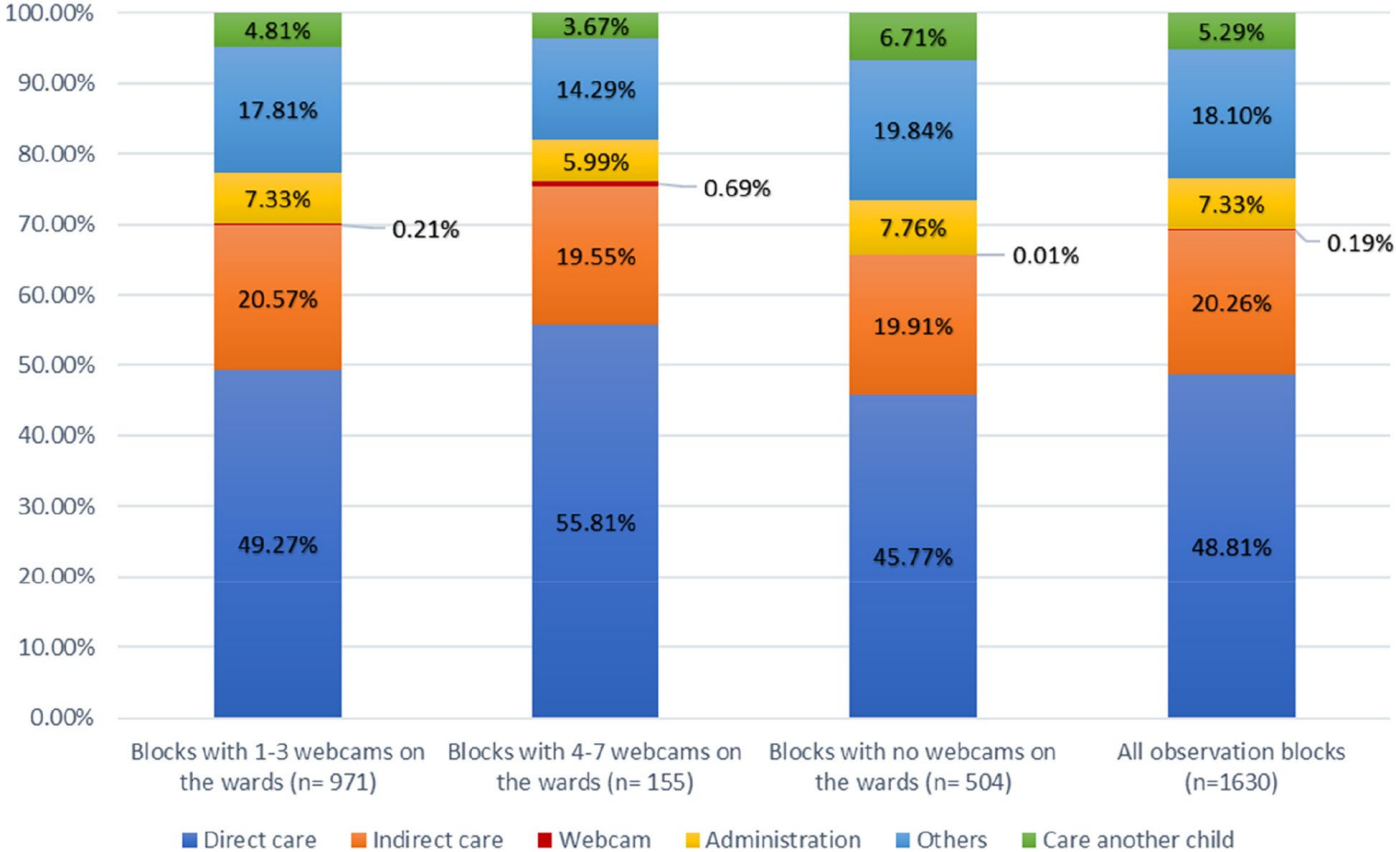
Distribution of time share per activity category

Table 2 shows the distribution of time for the observed activities and the average time per activity. In Fig. 1 the distribution of time share per activity category is presented. The webcam activities accounted for the lowest percentage of observed minutes in all observation block categories. The lowest mean time expenditure of all activity categories was also observed for the webcam activities, independently from the number of webcams on the wards.

**Table 2:** Distribution of observed time (in min.)

	Blocks with 1–3 webcams on the wards (*n* = 971)			Blocks with 4–7 webcams on the wards (*n* = 155)			Blocks with no webcams on the wards (*n* = 504)			All observation blocks (*n* = 1630)		
Activity	Total time	Share in %	Mean per activity	Total time	Share in %	Mean per activity	Total time	Share in %	Mean per activity	Total time	Share in %	Mean per activity
Direct care	35,543	49.27%	3.36	6,579	55.81%	2.45	17,369	45.77%	3.49	59,490	48.81%	3.26
Indirect care	14,835	20.57%	2.51	2,305	19.55%	1.78	7,555	19.91%	2.74	24,695	20.26%	2.48
Webcam	150	0.21%	0.70	82	0.69%	0.76	4	0.01%	0.37	236	0.19%	0.71
Administration	5,288	7.33%	3.91	706	5.99%	2.69	2,944	7.76%	3.87	8,938	7.33%	3.76
Others	12,848	17.81%	5.84	1,685	14.29%	5.30	7,530	19.84%	6.19	22,063	18.10%	5.91
Care another child	3,473	4.81%	3.34	433	3.67%	2.56	2,547	6.71%	3.81	6,452	5.29%	3.44
Total	72,137	100.00%	3.39	11,788	100.00%	2.44	37,949	100.00%	3.65	121,874	100.00%	3.34

The total time is the sum of all observed activities for each category (in min.). Based on this, the percentage time share was calculated. The last column shows the mean time per activity (in min.)

It can therefore be stated that the required time for webcam activities was relatively low. Nevertheless, a high number of webcam activities may lead to interruptions in the work process. Therefore, we analysed the blocks with a high number of webcam activities in more detail:

#### Analysis of the high-frequency webcam blocks

The mean of webcam activities per observation block was 2, therefore we defined high-frequency blocks as observation blocks who exceeded the mean (more than two webcam activities were observed per block).

21.08% of all webcam blocks were classified as high-frequency blocks. In these blocks, 46.85% of all observed webcam activities took place, and the webcam activities accounted – at 12.43% – for the third-highest share of all activities conducted after the direct and indirect care activities. We could see that the webcam activities take on average 13.02 s less than in the other webcam blocks.

Compared to the shift distribution of the webcam blocks with a maximum of two webcam activities, a higher share of blocks in the late and night shifts in the high-frequency blocks was observed (shift distribution high frequency blocks: early shift = 20.00%; late shift = 54.29%; night shift = 25.71% vs. blocks with max. two webcam activities: early shift = 38.93%; late shift = 47.33%; night shift = 13.74%).

The nurses in the blocks with a high level of webcam activity did not execute fewer other care activities (the webcam activities were not included in the calculation). In the high-frequency blocks, the nurses performed on average 31 activities per block, whereas the average activity level in the other webcam blocks is 27 activities; in all observation blocks (with a webcam at the incubator), it is 22 activities per block. If we compare the distribution of the activity categories between the high-frequency and low-frequency blocks, direct and indirect care tasks per block were nearly equally distributed; the deviations for all task categories (except the webcam activities) did not exceed 3.5%.

The average mean time per activity in all observation blocks was 3.34 min (see Table 2). The care tasks that were executed before the webcam activities were on average 74 s shorter in the high-frequency webcam blocks than in the other webcam blocks. The activities performed after the webcam task are 43.51 s shorter in the mean. To examine whether the nurses were more interrupted in the high-frequency webcam blocks due to the higher percentage of webcam activities, the activities before and after a webcam task were analysed in comparison to the webcam blocks with a maximum of two webcam activities. In the high-frequency blocks, for 64.1% of the webcam activities a different activity was performed before and after. In the other blocks, a different activity was performed 7.6% less frequently. This suggests that the nurses were not necessarily interrupted in their care tasks by the webcams, as they were doing something else before and after the webcam activity. The most frequently-executed activities after a webcam action in the high-frequency blocks were direct care (33.97%) and indirect care (29.49%). Therefore, we can state that no administrative tasks were inserted between webcam activities and the following activity. The nurses continued their workflow after a webcam activity. There was no clear indication for interruption due to the webcam-related activities.

### Questionnaire data

We collected 1,191 questionnaires from 65 nurses. The questionnaires in which the nurses did not complete the question about the additional workload caused by the webcams were excluded (*n* = 165). This results in 1,026 questionnaires from 62 nurses for the data analysis. The ANOVA showed that there were no significant differences between the frequency of completed questionnaires for each ward, so that the survey can be evaluated on aggregate (F (2,59) = 1.7, p = 0.1916). On average, each nurse completed 16 questionnaires over the study period. The extent to which the nurses had additional workloads due to the webcams was rated as no additional workload in 82.16% of the daily questionnaires (mean = 0.212, SD = 0.501, *n* = 1,026). The nurses stated in 73.78% of the questionnaires that they had no contact with a webcam on the observed day. The comparison between the rating of the nurses who did not have direct webcam contact (mean = 0.078, SD = 0.338, *n* = 757) and those who had contact with a webcam on the observation day (mean = 0.6, SD = 0.667, *n* = 265) showed that the additional workload caused by webcams is rated higher by the nurses with direct contact. Nevertheless, the workload was still rated very low even if the nurses had direct webcam contact.

The nurses who stated that they had direct webcam contact (*n* = 265) were asked to evaluate how much additional workload they perceived through different webcam-related tasks (see Table 3). The highest workload was recorded as a result of switching webcams on and off, as well as the adjustment of the webcams at the incubator. The least additional workload was perceived through telephoning with relatives about webcam-related topics.

**Table 3:** Perceived additional workload through webcam-related tasks

		N	Mean	SD
Webcam-related activities				
Switching the webcams on and off and adjusting them at the incubator	- no additional workload	120	0.573	0.577
	-rather low	124		
	-rather high	11		
	-very high	0		
	-missing	10		
Communication with relatives on the ward	- no additional workload	195	0.264	0.508
	-rather low	51		
	-rather high	8		
	-very high	0		
	-missing	11		
Communication with relatives through telephoning	- no additional workload	206	0.149	0.433
	-rather low	21		
	-rather high	7		
	-very high	0		
	-missing	31		
Technical problems	- no additional workload	225	0.150	0.473
	-rather low	20		
	-rather high	6		
	-very high	2		
	-missing	12		

### Ancillary analysis

The age group of participating nurses from 18 to 25 years was slightly overrepresented in comparison to all nurses working on the wards in the study (see Table 1). Therefore, we conducted additional analyses with regard to the age groups of the nurses. When comparing the time taken for webcam activity per age group, we observed that older nurses, on average, required marginally more time for these tasks (mean for nurses aged between 46 to 65 = 1.4 min per webcam activity vs. 0.62 min per webcam activity for nurses younger than 46). With regard to the additional perceived webcam workload, nurses aged between 46 to 55 years perceived the highest workload (mean = 0.279, SD = 0.451, *n* = 86), for nurses younger than 46 years the perceived workload was lower (mean = 0.217, SD = 0.516, *n* = 891). However, the lowest mean value was evaluated for the age group with nurses older than 55 years (mean = 0.02, SD = 0.143, *n* = 49).

## Discussion

To the best of our knowledge, this study was the first to investigate the objective workload in combination with the perceived workload of nurses due to the use of webcams on neonatal wards.

The data showed a small amount of additional work caused by webcam technology, in terms of observed activities and time. Only in 14.74% of the observation blocks with minimum one webcam on the wards were webcam activities by the nurses detected. Furthermore, the time required by the webcam system is very low (see Table 2). The results of the observation data align with the findings from the studies conducted by Chant et al. [18] and Kubicka et al. [6], indicating a low additional workload through the implementation of webcams. The insights from our observational data can mitigate the concern raised by nurses in previous studies [6, 19], that a significantly higher workload would arise.

The detailed analysis of the activities before and after the webcam, as well as the comparison of the number of activities in the high-frequency blocks, did not clearly indicate a higher workload or increased interruptions in the workflow due to the higher number of webcam activities. However, the shorter duration of activities before and after the webcam activities in these blocks could be an indication that more webcam activities could shorten other care activities. Therefore, our data analysis could not completely rule out the possibility of interruptions caused by the webcam-related activities. Chant et al. [18] and Kubicka et al. [6] for example evaluated interruptions of the nursing workflow due to technical issues or phone calls from the parents. This was not evident in our observational data, but the assessment of perceived additional workload caused by technical problems was ranked as the second-highest value in workload in the questionnaire data. There is scope for further research to analyse webcam related interruptions of the workflow.

For the increased observation of high-frequency observation blocks in late and night shifts several explanations are conceivable. Visiting hours in hospitals are often in the morning and early afternoon, so that the use of the webcam technology increases in the evening and night. It is also possible that the nursing staff in late and night shifts have more capacity to deal with the webcams out of self-interest.

The questionnaire data confirmed that the nursing staff themselves did not perceive a high additional workload due to the webcams. The activity of switching the webcams on and off, as well as aligning them, was rated by the nursing staff as requiring the most supplementary work. These tasks could be integrated into the work routine. For example, it would be possible to switch the webcams on and off at the beginning of each shift or at a certain fixed time. By integrating the plannable webcam activities into the workflow, the perceived workload for the nursing staff with direct webcam contact could be further reduced. Future research should evaluate strategies for a standardized integration of webcams into the nursing workflow.

The analysis of the questionnaires at individual nurse level showed that there are 27 of the 1,026 (2.63%) questionnaires in which the workload was rated high to very high. It is noticeable that 44.44% of these questionnaires were filled out by one person. The nurse completed a total of 21 questionnaires over the study period and indicated a rather high to very high additional workload in 57.14% of the questionnaires. It can be stated that, even if the aggregated workload is assessed as low, there may be individuals who perceive an additional burden in terms of their personal workload due to the webcams. Education of the nursing staff and implementation strategies into the workflow could be beneficial towards reducing the additional workload for the nursing staff on the individual level.

As the implementation of webcam technology has so far shown positive effects for the majority of patients and relatives, for example in terms of parental stress levels and feeling of reassurance  [5–7] it could also improve the interaction between parents and nurses on the ward. The concern of parents evaluated by Le Bris et al. [25] that a webcam could have negative impact on nurses and lead to more medical errors may be mitigated by our analysis, as both the objective time commitment and the perceived stress levels of the nurses were low.

Due to digitalization processes in the healthcare sector, technologies are constantly evolving, so further research must continue to monitor the relationship between nurses and technology.

### Limitations

Our findings should be interpreted in the context of the study’s limitations. Passive observation can lead to erroneous entries by the observer. This risk was kept as low as possible through the training of the observers and the use of observers with medical knowledge, as well as the technical function of noting incorrect information as a comment in the observation tool. Furthermore, the observation is associated with the possibility of nurses behaving differently than they would without being observed.

The methodological approach of the observational study was based on Langhammer et al. [21] and Sülz et al. [22]. They tested in their study the inter-rater reliability of the time recorded for each activity and found a high level of agreement between simultaneously collected data by pairs of observers. To establish an inter-rater reliability among the various observers in our study, all observers attended a digital information workshop about the study and received the same manual with guidelines on the observation and recording of activities.

With regard to the age structure on the wards, it can be stated that the study participants were younger. The age group from 18 to 25 years was slightly overrepresented in the study (see Table 1). Joshi et al. [7] ascertained that nurses with more work experience needed more time for webcam-related tasks. If we compare the different age groups in our study sample, we also noticed that older nurses on average needed marginally more time for these tasks.The younger nurses may have more intuitive access to technologies and were able to use them more quickly. The analysis of the questionnaire data with regard to the perceived webcam related workload supported this finding. The data showed that the highest mean was perceived from the nurses aged between 46 to 55 years. However, the lowest workload was perceived in the oldest age group, although it should be acknowledged that this age group comprises only two nurses.

The webcam related questions of the questionnaire were developed for the study itself and we pre-tested them with five nurses. Thus, only limited conclusions can be drawn regarding the validity and reliability.

The nurses completed the questionnaire several times; therefore, only the first questionnaires were independent and some bias and fatigue effect may occur due to repeated completion of questionnaires. Another aspect is that only nurses and no physicians were included in the study, but data published so far state that nurses are mainly responsible for the use of the webcam in the work processes [7, 12], and consequently most affected by the additional workload, .

### Implications for practice

The results indicated that, the use of webcam technology does not significantly increase the workload. Individual outliers may occur, but this does not result in increased workload or task reduction for nurses on the wards overall.

Therefore, from a managerial point of view there is no need to implement additional organisational measures, but managers need to raise awareness about the individual workload levels of nurses. Although the overall level of workload due to the webcam-related tasks was perceived as low, there may be nurses who are more stressed by the technology and need some support. Through regular staff appraisals, managers can evaluate the individual additional workload and take it into account accordingly. Supplementary education programs can be provided for nurses who have more difficulties with the technology.

## Conclusion

The recent pandemic situation has increased the importance of implementing new digital technologies in the hospital sector also. Multiple studies evaluated positive benefits of the webcam usage for neonatal patients on the parental perspective. For a successful implementation in standard care, it is highly important to consider the nursing perspective. The data analysis showed that the observed low workload is in line with the nurses’ perception of the additional webcam related workload. The evaluation indicated that a structured integration of plannable webcam-related activities into the workflow may be essential to reduce possible additional nursing workload. Thus, our results demonstrated that nurses may be encouraged to adopt and utilise the webcam technology, which could be a beneficial enhancement to neonatal care.

### Supplementary Information

Supplementary Material 1.

## Data Availability

The datasets used and analysed during the current study are available from the corresponding author on reasonable request.
