# Supplementary material for: Webcam technology on neonatal wards—examining the objective and subjective workload of nurses: a combined observational and survey study
Source: BMC Nurs. 2024 Jul 2;23:449. doi: 10.1186/s12912-024-02107-4 (PMC11218283; doi:10.1186/s12912-024-02107-4)
Supplement: Supplementary file 1 — Supplementary Material 1. [file 12912_2024_2107_MOESM1_ESM.docx]

Appendix to “Webcam technology on neonatal ward – Examining the objective and subjective workload of nurses: A combined observational and survey study”

Authors: Helena Sophie Müller, Michael Becker-Peth, Ludwig Kuntz, on behalf of Neo-CamCare

**Supplementary File 1: Questionnaire: Webcam activities during the work day**

| How would you rate the additional workload that the webcams have caused you personally today?  *(Please include communication with relatives related to the webcams).* |
| --- |
| □ no additional workload  □ rather low  □ rather high  □ very high |

| How much additional workload did you personally perceive today as a result of ...?  *(If you did not have contact with a webcam today, please only tick the box on the left)* | I did not have webcam contact today. | no additional workload | rather low | rather high | very high |
| --- | --- | --- | --- | --- | --- |
| … switching the webcams on and off and adjusting them. |  | □ | □ | □ | □ |
| … webcam related technical problems. |  | □ | □ | □ | □ |
| … communication with relatives about the webcam  *(e.g. counselling, problems with the application of the webcam, questions about the child's condition arising from webcam recordings)*  …on the ward. | □ | □ | □ | □ | □ |
| …through telephoning. |  | □ | □ | □ | □ |
